# Aflibercept Intervention in Experimental Branch Retinal Vein Occlusion Results in Upregulation of DnaJ Homolog Subfamily C Member 17

**DOI:** 10.1155/2021/6690260

**Published:** 2021-03-06

**Authors:** Lasse Jørgensen Cehofski, Anders Kruse, Alexander Nørgård Alsing, Benn Falch Sejergaard, Jonas Ellegaard Nielsen, Shona Pedersen, Danson Vasanthan Muttuvelu, Svend Kirkeby, Bent Honoré, Henrik Vorum

**Affiliations:** ^1^Department of Ophthalmology, Aalborg University Hospital, Aalborg, Denmark; ^2^Biomedical Research Laboratory, Aalborg University Hospital, Aalborg, Denmark; ^3^Department of Clinical Medicine, Aalborg University Hospital, Aalborg, Denmark; ^4^Department of Clinical Biochemistry, Aalborg University Hospital, Aalborg, Denmark; ^5^Department of Odontology, School of Dentistry, University of Copenhagen, Copenhagen, Denmark; ^6^Department of Biomedicine, Aarhus University, Aarhus, Denmark

## Abstract

Aflibercept is an inhibitor of vascular endothelial growth factor (VEGF) used to treat macular edema following branch retinal vein occlusion (BRVO). Despite well-documented efficacy, there is limited knowledge about proteome changes following aflibercept intervention in BRVO. Proteome changes may provide insights into mechanisms of action as well as aspects related to safety profile. In seven Danish Landrace pigs, BRVO was induced with a well-established experimental model of argon laser-induced BRVO. BRVO was induced in both eyes. Three days after the induced BRVO, aflibercept was injected intravitreally in the right eyes, while the left eyes received intravitreal isotonic saline water. Retinas were collected 15 days after the induced BRVO and analyzed with label-free quantification liquid chromatography tandem mass spectrometry (LFQ LC-MS/MS). Fourteen proteins were changed in expression following aflibercept intervention in the BRVO model. LFQ LC-MS/MS identified an upregulation of DnaJ homolog subfamily C member 17 (DNAJC17) (fold change = 6.19) and a modest downregulation of isoform 2 of the protein encoded by N-myc downstream regulated gene 2 (NDRG2) (fold change = 0.40). NDRG2 was unchanged by Western blotting. In the additional significantly regulated proteins, only discrete changes were observed (fold changes 0.52–1.59). Our study is the first to report an association between aflibercept intervention and the heat shock protein DNAJC17. Our results indicate that the role of heat shock proteins in the treatment of BRVO should be further explored.

## 1. Introduction

Branch retinal vein occlusion (BRVO) is the most frequent retinal vascular disease after diabetic retinopathy [1]. Occlusion of a retinal branch vein is known to cause retinal non-perfusion, retinal ischemia, and hypoxia, resulting in the release of vascular endothelial growth factor (VEGF) [2, 3]. Increased VEGF levels result in breakdown of the blood-retinal barrier leading to increased vascular permeability and formation of macular edema [1, 2, 4, 5]. Macular edema is a vision-limiting complication to BRVO which is effectively treated with intravitreal injections of VEGF inhibitors including bevacizumab, ranibizumab, and aflibercept [4, 6–9]. By neutralizing VEGF, anti-VEGF agents reduce retinal vascular permeability, leading to absorption of macular edema [4].

Bevacizumab is a monoclonal immunoglobulin G antibody and ranibizumab is a monoclonal antibody fragment (Fab) derived from bevacizumab. Bevacizumab and ranibizumab bind and neutralize all isoforms of VEGF-A [10]. Aflibercept is a fusion protein that contains the second immunoglobulin domain of VEGF receptor-1 (VEGFR-1) and the third immunoglobulin domain of VEGF receptor-2 (VEGFR-2) which are fused to the constant Fc domain of human immunoglobulin G1 [2, 11]. Aflibercept binds all isoforms of VEGF-A, VEGF-B, and placental growth factor [2, 11]. Using immunoprecipitation with aflibercept and retinal pigment epithelium (RPE) cell extracts, Kanda et al. [12] found that aflibercept also binds galectin-1 and suppresses galectin-1 mediated activation of VEGFR-2.

While novel mechanisms of action following aflibercept intervention have been discovered with immunoprecipitation, retinal proteome changes following aflibercept intervention in BRVO remain unstudied [13]. Therefore, the effect of aflibercept on retinal large-scale protein changes in BRVO remains largely unelucidated. The main objective of proteome studies is to study the entire set of proteins in a cell, tissue, or biofluid providing in depth information of how the given biological system works [13, 14]. Studying retinal proteome changes following aflibercept intervention may elucidate novel signaling pathways regulated by aflibercept and bring insights into novel mechanisms of action. Large-scale protein changes are important to uncover, for therapeutic, and also for safety reasons [13].

Protein changes in some ophthalmological conditions are best studied in appropriate animal models [15–17]. Animal models are almost always required to obtain retinal tissue exposed to BRVO [13]. Experimental BRVO may be induced with diathermia probes as well as argon laser [16, 18]. Experimental BRVO is effectively induced with diathermia probes, but this is an invasive approach which may potentially result in intraocular inflammation as well as changes in the proteome of the vitreoretinal interface [16, 18]. Laser-induced BRVO may be performed with or without an intravenous dye, for example, Rose Bengal. Rose Bengal facilitates induction of BRVO by absorbing the argon laser, but radiation of Rose Bengal may result in protein modifications such as conformational changes, oxidation of side chains, and unfolding [19].

For proteome studies, intravenous dye and invasive models with diathermia probes are best avoided. In the present study we tested aflibercept in a dye-free noninvasive porcine experimental model of argon laser-induced BRVO. The model results in classical findings observed in human BRVO such as flame-shaped hemorrhages, impaired drainage from the occluded vein, and breakdown of the blood-retinal barrier [16, 20–24]. The porcine eye is well-suited for studies of retinal vascular diseases, as the porcine retina is fully vascularized similar to the human retina [25]. Our previous studies have demonstrated that argon laser-induced experimental BRVO is a robust method in which BRVO does not re-canalize. The model is well suited for proteomic studies and can be used for relevant interventions including intravitreal anti-VEGF agents and dexamethasone implants [20, 21, 23]. We have previously tested ranibizumab, bevacizumab, and a dexamethasone implant on the model [20, 21, 24].

By combining the experimental BRVO model with advanced proteomic techniques, we provide insights into retinal proteome changes following aflibercept intervention in experimental BRVO.

## 2. Materials and Methods

The study was approved by the Danish Animal Experiments Inspectorate, permission no. 2019-15-0201-01651. The experiments were conducted in accordance with the guidelines published by the Institute for Laboratory Animal Research and in adherence to the ARVO Statement for the Use of Animals in Ophthalmic Research and Vision Research. Animals were housed under a 12 h light/dark cycle. Anesthesia, topical anesthesia with eye drops, and dilation of the pupils were performed as previously described [22].

### 2.1. Experimental Branch Retinal Vein Occlusion (BRVO) and Aflibercept Intervention

Experimental BRVO was induced in seven Danish Landrace pigs by using a well-established experimental model [16, 20, 21, 23, 24]. In both eyes, BRVO was induced close to the optic nerve head with a standard argon laser (532 nm), given by indirect ophthalmoscopy using a 20D lens. The laser energy was set to 400 mW with an exposure time of 550 ms. A total of 30–40 laser applications were used per occlusion. Experimental BRVO was considered successful when stagnation of venous blood and development of flame-shaped hemorrhages were observed. Interventions were performed three days after BRVO. Prior to interventions, both eyes were treated with povidone iodine eye drops (Skanderborg Pharmacy, Skanderborg, Denmark). Right eyes were then given an intravitreal injection of 0.05 mL aflibercept 40 mg/mL (Bayer, Leverkusen, Germany). Left eyes received an injection of 0.05 mL sodium chloride 9 mg/mL (NaCl) (B. Braun, Frederiksberg, Denmark). Following the injections, chloramphenicol ointment 1% (Takeda Pharma A/S, Taastrup, Denmark) was applied in both eyes. Fluorescein angiography was performed 5 days after BRVO was induced. Fifteen days after BRVO, the eyes were enucleated and immediately dissected on ice under a microscope. The anterior segment was removed by cutting 3.5 mm posteriorly to the limbus. The vitreous body was gently aspired into a 5 mL syringe. The neurosensory retina was peeled with tweezers from the RPE/choroid and stored at −80°C. The reproducibility of the BRVO model at the molecular level was tested in six additional Danish Landrace pigs. In these animals, BRVO (*n* = 6) was induced in the right eyes as described above. In the left eyes which served as controls (*n* = 6), identical laser burns were created with the same number of applications and laser energy as used in the right eyes without creating a BRVO. BRVO was confirmed with fluorescein angiography, and retinal samples were collected and stored as described above.

### 2.2. Sample Preparation for Mass Spectrometry

Samples intended for proteomic analysis by mass spectrometry were stored at −80°C until sample preparation for label-free tandem mass spectrometry was initiated. Eyes from six animals were used to compare the protein profile of BRVO + aflibercept (*n* = 6) vs. BRVO + NaCl (*n* = 6). The reproducibility of the BRVO model was tested by comparing BRVO (*n* = 6) and controls (laser only) (*n* = 6) with proteomic analysis.

Frozen samples were mixed with a lysis buffer consisting of 5% sodium deoxycholate (SDC) and 20 mM triethyl ammonium bicarbonate (TEAB) in a tissue-to-buffer ratio of approximately 1 : 20 (approximately 300 *µ*L). The samples were sonicated on ice with a Sonicator Q125 (QSonica LLC) with the following settings: amplitude 75%, three sonications for 59 seconds with 59-second rest between sonication runs. The samples were then incubated in a heating block with agitation (600 rpm) at 99°C for 5 min and were then allowed to chill slowly to room temperature. The extracts were centrifuged at 16,000*g* for 10 min, and supernatants were transferred to new 2 mL tubes.

The protein concentration of each sample was measured by infrared spectrometry using a Direct Detect Spectrometer (Merck KGaA, Darmstadt), as described in a recent article [26]. A volume of 5 *µ*L 200 mM tris 2-carboxyethyl phosphine (TCEP) and 20 mM TEAB solution was added to a 100 *µ*L sample, followed by incubation at 55°C for 1 h. Five *µ*L of freshly prepared 375 mM iodoacetamide was added to each sample followed by incubation for 30 min, at room temperature while being protected from light. The lysates were added to filter units (Microcon Centrifugal Filter Devices 30K from Millipore) and centrifuged at 14,000 *g* for 15 min. A volume of 100 *µ*L of digestion buffer (0.5% SDC, 20 mM TEAB) was added to the filter unit and centrifuged at 14,000*g* for 15 min. This step was performed twice. Afterwards, 2.5 *µ*g trypsin was added to each sample, followed by mixing at 600 rpm in a thermomixer for 1 min. The filter units were incubated overnight at 37°C. Filter units were transferred to new collection tubes and centrifuged at 14,000 *g* for 10 min. Another two centrifugation runs were performed with 100 *µ*L digestion buffer. An equal volume of ethyl acetate was added followed by addition of 10% trifluoroacetic acid (TFA) to a final concentration of 0.5% (vol/vol) of the final volume obtained after washing with digestion buffer. The samples were then shaken for 1 min, and centrifuged for 2 min at max speed, 14,000 rpm. The upper phase was removed and the lower phase was saved. Similar volumes of ethyl acetate were added two additional times, each time shaken, centrifuged, and finally removed. The samples were dried in a vacuum centrifuge, resuspended in 100 mM TEAB, and the peptide concentration was measured by fluorescence as previously described [24].

### 2.3. Quantitative Mass Spectrometry by Label-Free Quantitative Nanoliquid Chromatography Tandem Mass Spectrometry (LFQ LC-MS/MS)

Label-free quantification by nanoliquid chromatography tandem mass spectrometry was performed on an Orbitrap Fusion Tribrid mass spectrometer (Thermo Fisher Scientific Instruments, Waltham, MA, USA) coupled to a Dionex UltiMate^TM^ 3000 RSLC nano system. The instrument was equipped with an EasySpray™ ion source. Peptide separation by liquid chromatography was performed as previously described [24], with a few modifications. The analytical columns were 750 mm (750 mm × 75 *µ*m PepMap RSCL, C18, 2 *µ*m, 100 Å, Thermo Scientific) and a 4h gradient was made by mixing buffer A (99.9% water, 0.1% formic acid) with buffer B (99.9% acetonitrile, 0.1% formic acid). Mass spectrometry was performed with the Universal Method for label-free quantification. Two *µ*g of each sample was injected. Full scans were obtained in the Orbitrap at a scan range of 400–1500 m/z and a resolution of 120,000. Further settings included an automatic gain control target of 4 × 10^5^ and a maximum injection time of 50 ms. Precursor ions were isolated in the quadrupole. A collision-induced dissociation energy of 35% was applied. MS^2^ scans were detected in the ion trap with automatic gain control target of 2 × 10^3^ and a maximum injection time of 300 ms.

With MaxQuant software version 1.6.3.4 [27], raw data files generated from LFQ LC-MS/MS were searched against the Uniprot *Sus scrofa* database and the Uniprot *Homo sapiens* database as described in a previous work [24]. Unfiltered results from the database search are available in Appendix A.

### 2.4. Statistics

Mass spectrometry data was processed further with Perseus software version 1.6.2.3 [28] to remove poorly identified proteins as described in a previous article [22]. LFQ values were log2-transformed. Technical replicates were averaged. For successful protein identification, at least two unique peptides were required. Additionally, proteins were required to be successfully identified and quantified in at least 70% in each of the two groups compared. All proteins that were successfully identified and quantified are available in Appendix B. Statistical analysis by Student's *t*-test was performed in Perseus and proteins were considered statistically significantly regulated if *p* < 0.01. Volcano plots were created in STATA 16.0 (StataCorp, College Station, TX, USA) by plotting −log(*p* value) against log2-transformed LFQ abundance ratios.

### 2.5. Western Blotting

Western blotting was performed as previously described [22], with the exception that protein concentrations were adjusted by adding 5% SDC with 20 mM TEAB and that expression of the protein under validation was normalized using a polyclonal rabbit anti-beta tubulin antibody 1 : 500 (AB6046, Abcam, Cambridge, UK). NDRG2 was quantified with a polyclonal rabbit antibody directed at NDRG2 1 : 500 (MBS8207067, MyBioSource, San Diego, CA, USA). Statistical analysis was performed by Student's *t*-test on log-transformed densitometric data.

### 2.6. Immunohistochemistry

Eyes from one animal were used to compare BRVO + aflibercept (*n* = 1) with BRVO + NaCl (*n* = 1). Immunohistochemistry was performed as previously described [24]. Briefly, retinal samples were fixated in a fixative of 99% ethanol (three parts) and glacial acetic acid (one part) for 5 h and stored in a 0.1 M phosphate buffer solution at 4°C until further use. Immunohistochemistry was performed with the same anti-NDRG2 antibody as described above for Western blotting (AB8069, Abcam, Cambridge, UK). For immunohistochemistry, the antibodies were diluted in PBS + 0.3% Triton X100 reaching a dilution of 1 : 500. Retinal sections were incubated overnight at 4°C and processed with EnVision DakoCytomation DAB.

## 3. Results

### 3.1. Evaluation of Experimental Branch Retinal Vein Occlusion

**Figure 1:**
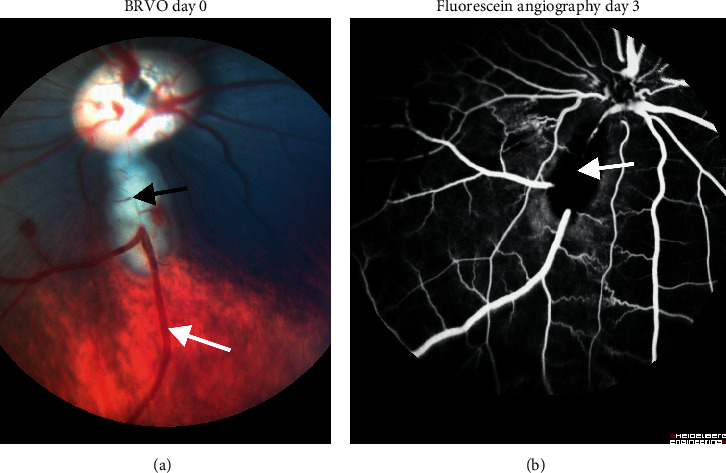
Experimental branch retinal vein occlusion (BRVO). (a) Fundus image of BRVO taken approximately 10 minutes after BRVO was induced in the inferior retina. Black arrow: site of occlusion. White arrow: dilated vein upstream of occlusion. (b) Validation of BRVO by fluorescein angiography five days after BRVO. No passage of fluorescein through the occluded vein is observed. Shunting to adjacent veins is observed. White arrow: site of occlusion.

Experimental BRVO was successfully induced based on development of venous dilation and flame-shaped hemorrhages (Figure 1(a)). Fluorescein angiography performed five days after BRVO showed no recanalization of the occluded veins in any of the animals (Figure 1(b)).

Proteomic analysis by mass spectrometry confirmed a good reproducibility of the BRVO model at the molecular level. Protein changes in the BRVO model were consistent with protein changes that were previously observed in the model (Appendix C) [23].

### 3.2. Proteome Changes following Aflibercept Intervention

High contents of aflibercept components were observed in retinas treated with the compound. The VEGFR-1 immunoglobulin domain of aflibercept was only identified and quantified in retinas treated with aflibercept. The VEGFR-1 immunoglobulin domain was identified as UniProtID P17948. A high content of the VEGFR-2 immunoglobulin domain of aflibercept was observed in retinas treated with aflibercept (fold change = 64.64; *p* value = 0.000016) (UniProtID P35968) (Table 1).

**Figure 2:**
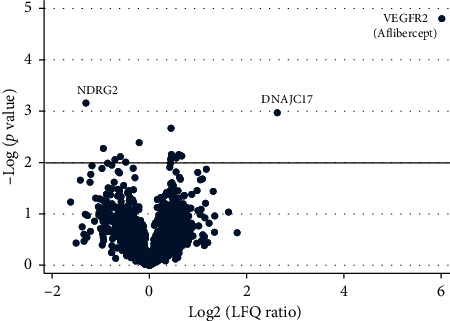
Volcano plot. Quantitative data from label-free quantification liquid chromatography tandem mass spectrometry (LFQ LC-MS/MS) were measured as label-free quantification (LFQ) values. Log2 LFQ ratios (aflibercept/NaCl) are plotted on the *x*-axis. On the *y*-axis, −log *p* value refers to the logarithmized *p* value from the *t*-test used to test if a protein was significantly regulated. The black horizontal line denotes a significance level corresponding to *p*=0.01. The proteins with the largest changes are indicated in the plot.

**Figure 3:**
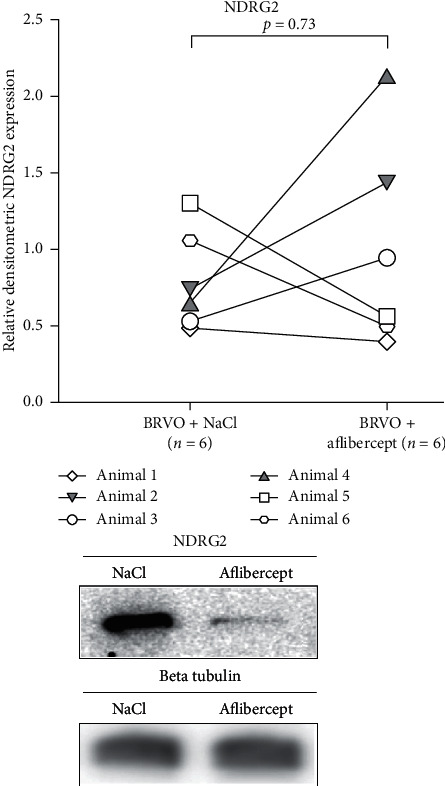
Proteomic analysis by mass spectrometry revealed a downregulation of NDRG2 in the BRVO model following aflibercept intervention. However, the downregulation of NDRG2 was not sufficiently large to be confirmed with Western blotting (*p*=0.73).

**Figure 4:**
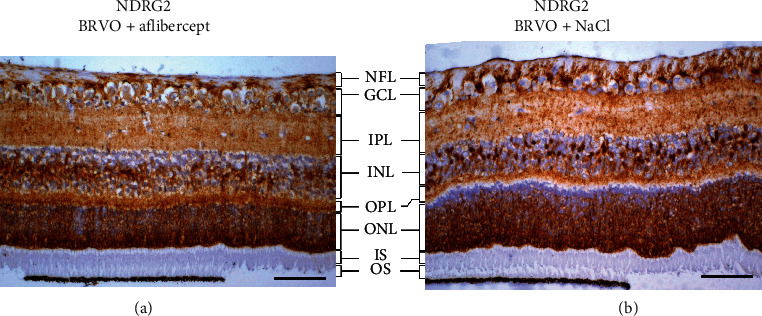
(a) and (b), immunohistochemistry showed a slight downregulation of NDRG2 in the retinal ganglion cell layer and the inner nuclear layer following aflibercept intervention in BRVO. Scale bar = 31 *µ*m. Reaction color: brown. ILM: inner limiting membrane; NFL: nerve fiber layer; GCL: ganglion cell layer; IPL: inner plexiform layer; INL: inner nuclear layer; OPL: outer plexiform layer; ONL: outer nuclear layer; IS: inner segment; OS: outer segment.

Fourteen proteins were statistically significantly changed following aflibercept intervention in BRVO (Table 1) (Figure 2). An upregulation of DnaJ homolog subfamily C member 17 (DNAJC17) was identified following aflibercept intervention in BRVO (fold change = 6.19; *p*=0.0011). A slight downregulation of isoform 2 of the protein of N-myc downstream regulated gene 2 (NDRG2) following aflibercept intervention was observed with LFQ LC-MS/MS (fold change = 0.40; *p*=0.00070). The modest downregulation of NDRG2 was not confirmed with Western blotting (fold change = 1.17; *p*=0.73) (Figure 3). Immunohistochemistry revealed some decreased staining for NDRG2 following aflibercept intervention in BRVO (Figures 4(a) and 4(b)).

Decreased staining for NDRG2 was observed in the nerve fiber layer, the ganglion cell layer, and the inner nuclear layer. In the additional proteins that were regulated following aflibercept intervention, fold changes ranged from 0.52 to 1.59 (Table 1) (Figure 2).

## 4. Discussion

### 4.1. Experimental Model of Branch Retinal Vein Occlusion

There are a number of models of experimental BRVO that are available for research purposes [18, 29]. The argon laser-induced BRVO model used in our study is well-suited for proteomic studies as the model is noninvasive [16, 20, 23]. Alternatively, invasive models with, for example, diathermia probes may affect the proteome of the vitreoretinal interface which renders invasive models less suitable for proteomic studies [16].

Full occlusion of a retinal branch vein may be difficult to achieve in experimental BRVO models and recanalization may be a problem [16, 29]. In the presented study, we demonstrated that recanalization did not occur in the BRVO model.

In most cases, BRVO occurs at sites of arteriovenous crossing where the retinal vein is compressed by a retinal artery that has thickened due to years of hypertension and arteriosclerosis [1, 30]. Compression of a retinal vein by an arteriosclerotic artery is not possible to induce in animal models of BRVO [13, 29]. With no underlying arteriosclerotic disease, pigs have a faster recovery from BRVO than humans [23]. The time frame of 14 days following experimental BRVO is considered suitable for the study of BRVO and interventions, as the model translates well into findings observed in human BRVO within this period [21, 23].

Previous reports have shown that anti-VEGF injections can have some effect in the untreated fellow eye [31, 32]. However, results from our previous studies of the BRVO model have indicated that anti-VEGF agents reach high retinal concentrations with high concentration differences between treated and nontreated eyes [20, 21]. Therefore, the fellow eye was considered suitable as a control.

### 4.2. Aflibercept Intervention in Experimental BRVO

High retinal contents of VEGFR-1 and VEGFR-2 immunoglobulin domains of aflibercept were measured in retinas treated with aflibercept. Thus, our data indicates that very high levels of aflibercept are reached within 14 days of treatment. Only 14 proteins were statistically significantly changed in content following aflibercept intervention. Based on our proteomic data, we did not find that aflibercept regulated multiple proteins in the BRVO model. In terms of safety profile, it may be an important observation that aflibercept did not cause changes in multiple signaling pathways that may have unwanted side effects. Unlike intravitreal dexamethasone [24], aflibercept did not regulate a broad spectrum of proteins indicating that dexamethasone implants have a wider mechanism of action than aflibercept. Knowledge about the spectrum of proteins regulated by aflibercept is important when making decisions about adequate therapy.

The proteins with largest regulations included DNAJC17 and possibly NDRG2. DNAJC17 is a heat shock protein family member known to be involved in splicing-related processes [33], but the function of DNAJC17 remains largely unknown [33]. DNAJC17 belongs to a class of cofactors called J-proteins. J-proteins are cofactors of 70 kilodalton heat shock proteins (Hsp70s). The so-called J-domain of the J-proteins stimulates ATPase activity of Hsp70s. Mutations in the DNAJC17 gene have been reported to be associated with retinitis pigmentosa and hypogammaglobulinemia [34]. A mutation in the DNAJC17 gene has also been described in patients with essential thrombocytopenia [35]. To the best of our knowledge, our study is the first to describe an association between aflibercept intervention and DNAJC17. Further investigation is needed in order to establish the role of DNAJC17 in the treatment of BRVO.

Immunohistochemistry showed slightly reduced staining for NDRG2 in the nerve fiber layer, the ganglion cell layer, and the inner nuclear layer following aflibercept intervention. As the change in NDRG2 was small, Western blotting may not have been sufficiently sensitive to confirm the regulation of NDRG2. NDRG2 belongs to the family proteins encoded by N-Myc downstream-regulated genes (NDRGs) and is expressed in many cellular compartments including cytoplasm, cell membrane, and nuclei [36]. NDRG2 is markedly upregulated in hypoxic conditions [37, 38]. There is only limited knowledge about the role of retinal NDRG2 and further studies are required to determine if retinal NDRG2 is a hypoxia sensitive protein in BRVO.

Changes in the additional significantly regulated proteins were small, ranging within fold changes of 0.52–1.59. Changes in content were minimal in most of the proteins. Moderate changes in content were observed in Bcl-2-like protein 13 (fold change = 1.59), inosine-5-monophosphate dehydrogenase 2 (fold change = 1.52), 5-3 exoribonuclease 2 (fold change = 1.47), and MICOS complex subunit MIC60 (MICOS) (fold change = 0.52). Bcl-2-like protein 13 is known to be associated with removal of damaged mitochondria [39]. Inosine-5-monophosphate dehydrogenase 2 is involved in de novo synthesis of guanine nucleotides and a regulator of cell growth [40]. 5-3 exoribonuclease 2 is a promoter of RNA polymerase II transcription [41]. MICOS complex subunit MIC60 is a component of the mitochondrial contact site and cristae organizing system (MICOS) complex. The MICOS complex is involved in maintenance of mitochondrial structure, lipid metabolism, and protein import [42].

In our previous bevacizumab study, we identified an increased retinal content of transthyretin following bevacizumab intervention in the BRVO model [20]. In the present aflibercept study, we identified an increased level of transthyretin following aflibercept intervention, but the upregulation was not statistically significant (*p*=0.15; fold change = 2.34). Other proteins that were regulated by bevacizumab were not identified with mass spectrometry in the present study.

## 5. Conclusions

With advanced proteomics techniques, we aimed at identifying large-scale retinal protein changes following aflibercept intervention in experimental BRVO. Overall, very few proteins were regulated following aflibercept intervention, and modest changes were observed in most of the regulated proteins. Our study identified an upregulation of the heat shock protein DNAJC17 following aflibercept intervention in experimental BRVO. To the best of our knowledge, an association between DNAJC17 and aflibercept intervention has not previously been described. Further studies will be required to shed light on the function of retinal DNAJC17, and a potential association with VEGF. Our findings also indicate that the role of heat shock proteins in BRVO warrants further study. A downregulation of NDRG2 was observed in experimental BRVO treated with aflibercept. Further studies are necessary to uncover the biological role of retinal NDRG2. Based on our proteomic data, aflibercept did not regulate multiple signaling pathways. In terms of safety profile, it is a relevant observation that aflibercept did not cause changes in multiple signaling pathways that may have unwanted side effects. In retinas treated with aflibercept, high retinal levels of VEGFR-1 and VEGFR-2 immunoglobulin domains of aflibercept were observed indicating that a high aflibercept concentration was reached within 14 days of aflibercept treatment.

## Figures and Tables

**Table 1:** Proteins which were regulated following aflibercept intervention in experimental BRVO.

Protein ID	Protein name	*p* value	Fold change
P35968	Vascular endothelial growth factor receptor 2 (aflibercept)	0.000016	64.64
Q9NVM6	DnaJ homolog subfamily C member 17	0.0011	6.19
Q9BXK5-2	Isoform 1 of Bcl-2-like protein 13	0.0075	1.59
P12268	Inosine-5-monophosphate dehydrogenase 2	0.0070	1.52
Q9H0D6-2	Isoform 2 of 5-3 exoribonuclease 2	0.0082	1.47
Q9UPQ0-10	Isoform 10 of LIM and calponin homology domains-containing protein 1	0.0070	1.37
Q8WZA9	Immunity-related GTPase family *Q* protein	0.0022	1.36
P63208-2	Isoform 2 of S-phase kinase-associated protein 1	0.0086	1.35
Q6Q7J2	Rab GDP dissociation inhibitor beta	0.0041	0.86
P62191	26S proteasome regulatory subunit 4 subunit 4	0.0098	0.71
P20585	DNA mismatch repair protein Msh3	0.0077	0.66
Q29195	60S ribosomal protein L10	0.0088	0.61
Q16891-3	Isoform 3 of MICOS complex subunit MIC60	0.0053	0.52
Q9UN36-2	Isoform 2 of protein NDRG2	0.00070	0.40

## Data Availability

The data used to support the findings of this study are included within the supplementary materials.

## Supplementary Materials

Supplementary Materials Appendix A: unfiltered results from database search. Appendix B: all successfully identified and quantified proteins. Appendix C: test of reproducibility of the BRVO model at the molecular level.
