# Supplementary material for: Aflibercept Intervention in Experimental Branch Retinal Vein Occlusion Results in Upregulation of DnaJ Homolog Subfamily C Member 17
Source: J Ophthalmol. 2021 Mar 6;2021:6690260. doi: 10.1155/2021/6690260 (PMC7960061; doi:10.1155/2021/6690260)
Supplement: Supplementary Materials — Appendix A: unfiltered results from database search. Appendix B: all successfully identified and quantified proteins. Appendix C: test of reproducibility of the BRVO model at the molecular level. [file 6690260.f1.zip › Appendix C (1).pdf]

Supplementary table 3 – significantly regulated proteins in BRVO vs. control

Protein ID	Protein name	Gene name	P-value	Ratio BRVO/control
Q14315-2	Isoform 2 of Filamin-C	FLNC	0.00019	6.03
Q8MJ14	Glutathione peroxidase 1	GPX1	0.0064	2.94
P08835	Serum albumin	ALB	0.0044	2.70
P31950	Protein S100-A11	S100A11	7.92×10^{-5}	2.60
P14287	Osteopontin	SPP1	0.0043	2.60
P37802	Transgelin-2	TAGLN2	0.0022	2.50
P02543	Vimentin	VIM	0.00021	2.45
Q863Z0	Proteasome activator complex subunit 2	PSME2	0.0046	2.42
P19620	Annexin A2	ANXA2	0.00053	2.41
P08132	Annexin A4	ANXA4	0.0028	2.41
Q9BX66-9	Isoform 9 of Sorbin and SH3 domain-containing protein 1 domain-containing protein 1	SORBS1	0.0047	2.36
P19619	Annexin A1	ANXA1	0.0016	2.31
Q9H4A4	Aminopeptidase B	RNPEP	0.0013	2.30
P01846	Ig lambda chain C region		0.00098	2.23
P14136	Glial fibrillary acidic protein	GFAP	0.0064	2.18
P08758	Annexin A5	ANXA5	0.0031	1.98
P21333-2	Isoform 2 of Filamin-A	FLNA	0.00024	1.90
Q7M2W6	Alpha-crystallin B chain	CRYAB	0.0032	1.90
P13797	Plastin-3	PLS3	0.0071	1.86
P83731	60S ribosomal protein L24	RPL24	0.0089	1.80
O00629	Importin subunit alpha-3	KPNA4	0.0096	1.80
P50995-2	Isoform 2 of Annexin A11	ANXA11	0.0028	1.77
Q96J01-2	Isoform 2 of THO complex subunit 3	THOC3	0.002	1.75
P12814	Alpha-actinin-1	ACTN1	7.16×10^{-5}	1.72
Q8NEU8	DCC-interacting protein 13-beta	APPL2	0.0056	1.61
Q13642-1	Isoform 1 of Four and a half LIM domains protein 1	FHL1	0.0019	1.58
P35579	Myosin-9	MYH9	0.0067	1.53
Q9Y2J0-2	Isoform 2 of Rabphilin-3A	RPH3A	0.0035	1.52
P82460	Thioredoxin	TXN	0.0059	1.45
O15031	Plexin-B2	PLXNB2	0.0051	1.35
O43707	Alpha-actinin-4	ACTN4	0.0049	1.33
O43347	RNA-binding protein Musashi homolog 1	MSI1	0.0017	1.28
Q14558	Phosphoribosyl pyrophosphate synthase-associated protein 1	PRPSAP1	0.002	0.81
P09104	Gamma-enolase	ENO2	0.0055	0.81
P13591-4	Isoform 4 of Neural cell adhesion molecule 1	NCAM1	0.005	0.79
O95782-2	Isoform B of AP-2 complex subunit alpha-1	AP2A1	0.0072	0.79
Q02218|O	2-oxoglutarate dehydrogenase,	OGDH	0.0031	0.78
P21281	V-type proton ATPase subunit B, brain isoform	ATP6V1B2	0.00016	0.75
Q99962	Endophilin-A1	H3GL2	0.0056	0.75
Q8MIZ3	m7GpppX diphosphatase	DCPS	0.0043	0.72

P62760	Visinin-like protein 1	VSNL1	0.0068	0.66
Q99623-2	Isoform 2 of Prohibitin-2	PHB2	0.002	0.65
O97580	Succinate--CoA ligase [ADP-forming] subunit beta, mitochondrial (Fragment)	SUCLA2	0.00098	0.64
Q9UHD9	Ubiquilin-2	UBQLN2	0.0023	0.64
P43007	Neutral amino acid transporter A	SLC1A4	0.0035	0.57
P10745	Retinol-binding protein 3	RBP3	0.0077	0.55
Q8HZV3	Transferrin receptor protein 1	TFRC	0.0024	0.54
Q13884-2	Isoform 2 of Beta-1-syntrophin	SNTB1	0.0083	0.51
